# Follow-up infarct volume as a mediator of endovascular treatment effect on functional outcome in ischaemic stroke

**DOI:** 10.1007/s00330-018-5578-9

**Published:** 2018-07-09

**Authors:** K. C. J. Compagne, A. M. M. Boers, H. A. Marquering, O. A. Berkhemer, A. J. Yoo, L. F. M. Beenen, R. J. van Oostenbrugge, W.H. van Zwam, Y. B. W. E. M. Roos, C. B. Majoie, A. C. G. M. van Es, A. van der Lugt, D. W. J. Dippel, H. Lingsma, for the MR CLEAN Investigators

**Affiliations:** 1 Department of Radiology and Nuclear Medicine, Erasmus MC University Medical Center, PO Box 2040 3000, CA Rotterdam, The Netherlands; 2 Department of Neurology, Erasmus MC University Medical Center, Rotterdam, The Netherlands; 3 Department of Radiology and Nuclear Medicine, Academic Medical Center (AMC), Amsterdam, The Netherlands; 4 Biomedical Engineering and Physics, Academic Medical Center (AMC), Amsterdam, The Netherlands; 5 Department of Robotics and Mechatronics, University of Twente, Enschede, The Netherlands; 6 Division of Neurointervention, Texas Stroke Institute, Dallas-Fort Worth, TX USA; 7 Department of Neurology, Maastricht University Medical Center, Maastricht, The Netherlands; 8 Cardiovascular Research Institute Maastricht, Maastricht, The Netherlands; 9 Radiology, Maastricht University Medical Center, Maastricht, The Netherlands; 10 Neurology, Academic Medical Center (AMC), Amsterdam, The Netherlands; 11 Department of Public Health, Erasmus MC University Medical Center, Rotterdam, The Netherlands

**Keywords:** Stroke, Thrombectomy, Causality, Outcome, Biomarkers

## Abstract

**Objective:**

The putative mechanism for the favourable effect of endovascular treatment (EVT) on functional outcome after acute ischaemic stroke is preventing follow-up infarct volume (FIV) progression. We aimed to assess to what extent difference in FIV explains the effect of EVT on functional outcome in a randomised trial of EVT versus no EVT (MR CLEAN).

**Methods:**

FIV was assessed on non-contrast CT scan 5–7 days after stroke. Functional outcome was the score on the modified Rankin Scale at 3 months. We tested the causal pathway from intervention, via FIV to functional outcome with a mediation model, using linear and ordinal regression, adjusted for relevant baseline covariates, including stroke severity. Explained effect was assessed by taking the ratio of the log odds ratios of treatment with and without adjustment for FIV.

**Results:**

Of the 500 patients included in MR CLEAN, 60 died and four patients underwent hemicraniectomy before FIV was assessed, leaving 436 patients for analysis. Patients in the intervention group had better functional outcomes (adjusted common odds ratio (acOR) 2.30 (95% CI 1.62–3.26) than controls and smaller FIV (median 53 vs. 81 ml) (difference 28 ml; 95% CI 13–41). Smaller FIV was associated with better outcome (acOR per 10 ml 0.60, 95% CI 0.52–0.68). After adjustment for FIV the effect of intervention on functional outcome decreased but remained substantial (acOR 2.05, 95% CI 1.44–2.91). This implies that preventing FIV progression explains 14% (95% CI 0–34) of the beneficial effect of EVT on outcome.

**Conclusion:**

The effect of EVT on FIV explains only part of the treatment effect on functional outcome.

**Key Points:**

*• Endovascular treatment in acute ischaemic stroke patients prevents progression of follow-up infarct volume on non-contrast CT at 5*–*7 days.*

*• Follow-up infarct volume was related to functional outcome, but only explained a modest part of the effect of intervention on functional outcome.*

*• A large proportion of treatment effect on functional outcome remains unexplained, suggesting FIV alone cannot be used as an early surrogate imaging marker of functional outcome.*

**Electronic supplementary material:**

The online version of this article (10.1007/s00330-018-5578-9) contains supplementary material, which is available to authorized users.

## Introduction

In 2015, endovascular treatment (EVT) was shown to be effective in improving functional outcome in patients with ischaemic stroke due to intracranial large vessel occlusion [1]. Secondary outcome analyses of the randomised clinical trials also indicated significantly smaller infarct volumes at follow-up imaging in patients who were allocated to the intervention group [2, 3]. Studies have suggested that follow-up infarct volume (FIV) could be a useful early outcome measure [4–6].

FIV as a surrogate outcome is a well-quantifiable measure and therefore less sensitive to interobserver variability compared to clinical assessment of functional outcome such as the modified Rankin Scale score at 90 days [7, 8]. Also, FIV measurements can be assessed relatively easily and semi-automatically after treatment on non-contrast computed tomography (NCCT) or magnetic resonance imaging (MRI) scans [9, 10]. A moderate correlation between FIV and clinical outcome has been demonstrated [11]. FIV has been suggested as a primary endpoint in late phase II clinical trials, which are intended to demonstrate an indication of therapeutic effect in promising novel treatments. Assessment of functional outcome as a clinical endpoint requires prolonged follow-up. An early surrogate marker could therefore be more feasible in clinical trials, and limit loss to follow-up [12, 13].

A recent post hoc study demonstrated that the beneficial effect of EVT on functional outcome could be explained by preventing progression of FIV, suggesting that the effect of intervention on functional outcome is mediated by FIV [6]. Formal testing of such a mechanism requires a causal mediation model to estimate the extent to which the treatment effect is explained by a mediator [14]. This is usually expressed as a proportion of the original treatment effect. In the context of testing a mediator as a surrogate marker, the Prentice criteria have been proposed to formally test for a causal relation between surrogate and clinical endpoints [15]. This analytical approach for estimating the causal effect of FIV on functional outcome has not yet been fully reported for EVT in acute ischaemic stroke and the extent to which the beneficial effect of intervention on functional outcome can be explained by difference in FIV is not yet known [16]. Understanding the causal pathway of this relation may provide further insight and may help in developing surrogate markers of functional outcome after EVT, and shed further light on outcome predictors that can be used for future stroke trials. The aim of this study was to assess whether and to what extent FIV on NCCT at 5–7 days’ follow-up is a mediator of the effect of intervention on functional outcome in acute ischaemic stroke patients.

## Material and methods

### Patients

In this post-hoc analysis, we used data from the Multicenter Randomised Clinical Trial of Endovascular Treatment for Acute Ischemic Stroke in The Netherlands (MR CLEAN), which was performed at 16 Dutch stroke centres [2]. This randomised trial investigated the effect of EVT plus usual care (intervention) versus usual care only (control). In both treatment groups, administration of intravenous alteplase was allowed before randomisation. Patients had a minimal score of 2 on the National Institutes of Health Stroke Scale (NIHSS) at baseline and a radiologically confirmed proximal intracranial arterial occlusion of the anterior circulation. Follow-up imaging by computed tomography (CTA) or magnetic resonance angiography (MRA) was done at 24 h to assess endovascular recanalisation. After 5–7 days, a (NCCT) scan was acquired to assess FIV and haemorrhagic transformation. Institutional review board approval and written informed consent from all patients were obtained [2]. In the present study, patients were excluded if they died before the follow-up NCCT scans at 5–7 days or in case no NCCT was acquired before hemicraniectomy.

### Measures

FIVs at 5–7 days’ follow-up were semi-automatically segmented with the use of validated in-house- developed software based on intensity region growing algorithm [17]. Placement of seed points for initiating region growing in infarcted areas was done by an experienced radiologist to overcome selection of older infarctions. Segmentations were inspected and if necessary manually adjusted by two observers, who were blind to treatment allocation, as previously described [9]. FIV was calculated by multiplying the number of voxels with the voxel size. The semi-automated segmentations were highly correlated (Pearson’s correlation coefficient of 0.98) to reference manual measurements [17]. Two examples of the semi-automatic segmentation process are shown in Fig. 2. Post-treatment functional outcome was measured on the modified Rankin Scale (mRS) at 90 days and was assessed in a standardised telephone interview by a single investigator and validated by blinded assessors. The mRS is a 7-point scale ranging from 0 (no symptoms) to 6 (death).

**Fig. 1:**
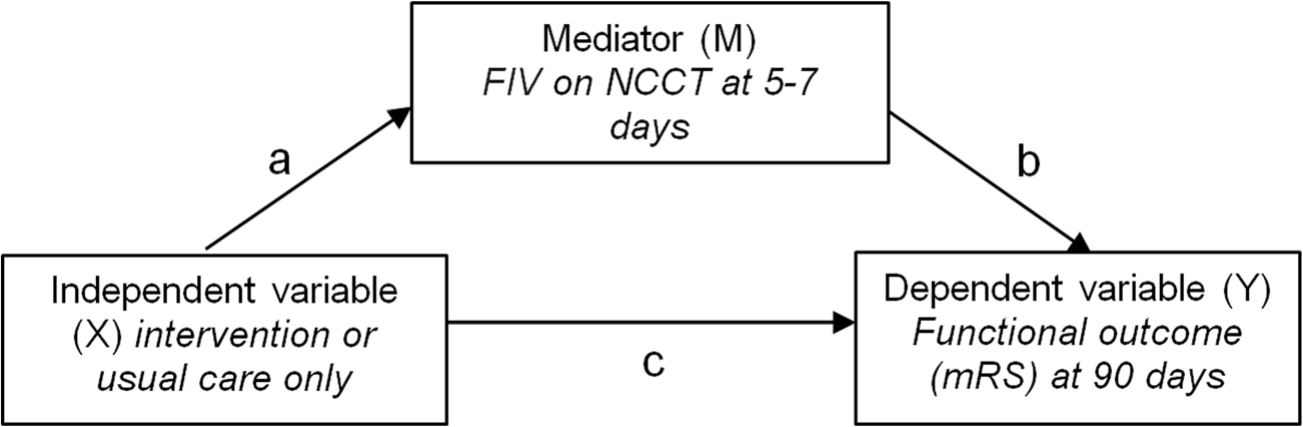
Causal diagram showing the mediation model. Arrows are the causal direction or possible association

**Fig. 2:**
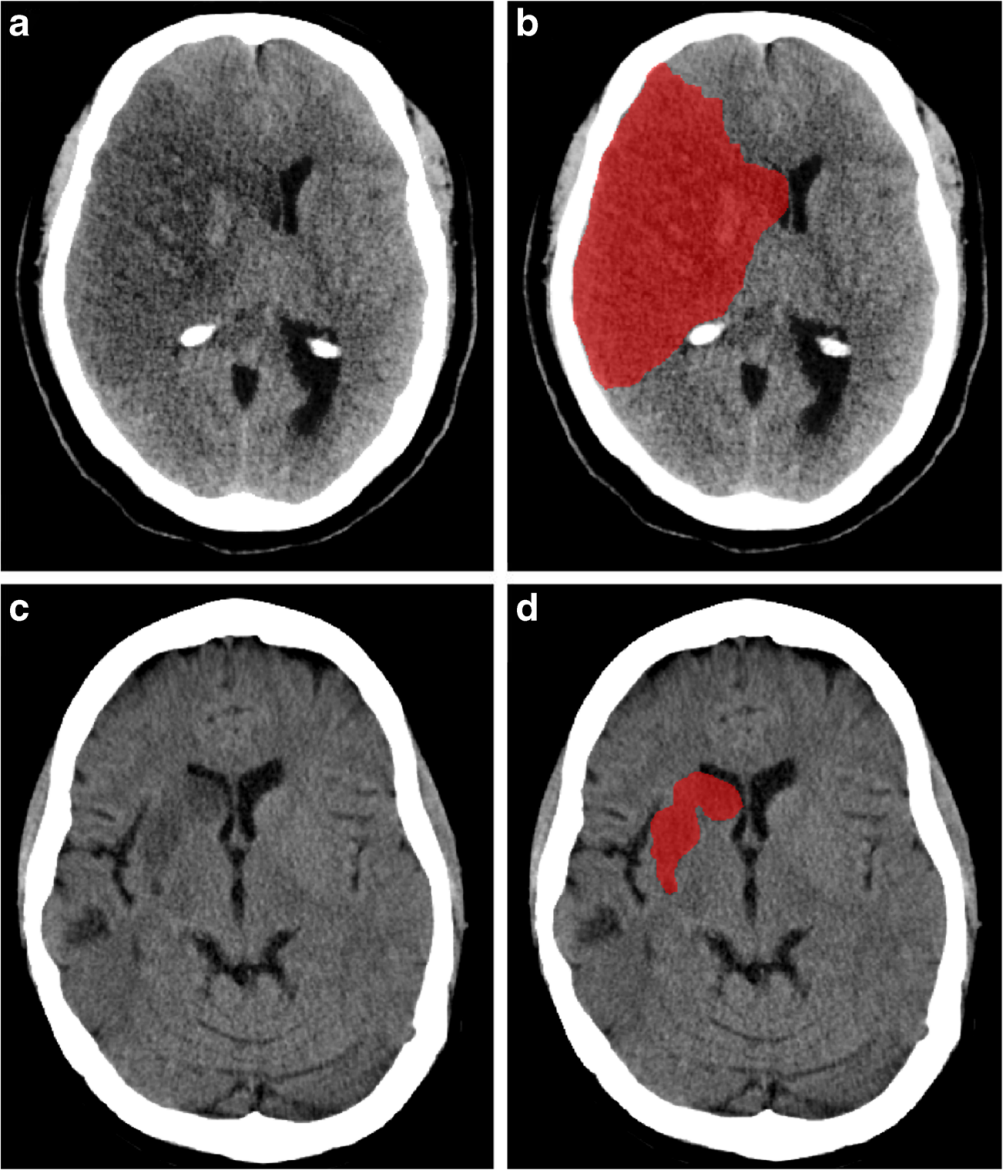
Case examples of follow-up infarct (FIV) segmentation on non-contrast CT, acquired between 5 and 7 days after onset. (**a** and **b**) A 56-year-old male with right-sided M1 occlusion. FIV was 292 ml and this patient was severely disabled at 90 days (mRS 5). (**c** and **d**) A 45-year-old female with right-sided M1 occlusion. FIV was 10 ml and the patient showed no significant disability at 90 days, despite some symptoms (mRS 1)

### Statistical analysis

The confidence interval of the difference of measured median FIVs between both treatment groups was tested by bootstrapping with 1,000 replications. Statistical testing of a mechanism or pathway requires a mediation model [14]. Rather than a direct causal relationship between the independent variable (intervention or control group) and the dependent variable (functional outcome), a mediation model proposes that the independent variable influences the mediator variable (FIV on NCCT at 5–7 days), which in turn influences the dependent variable (Fig. 1).

Three requirements must be met to prove a true mediation relationship [14]:

1. The independent variable must be a significant predictor of the dependent variable (Fig. 1, pathway C).
2. The independent variable is a significant predictor of the mediator (Fig. 1, pathway A).
3. The mediator is a significant predictor of the dependent variable, while controlling for the independent variable. In other words: when treatment allocation and FIV are combined in one model to predict functional outcome (i.e. pathway A-B), FIV should still be a significant predictor, while the effect of intervention should be strongly reduced (compared to the unadjusted effect). This step is needed to prove that the effect goes (partly) through pathway A-B instead of C (Fig. 1).

According to the Prentice criteria, FIV must completely account for the net effect of intervention to be a perfect surrogate, meaning that in step 3 the effect of intervention on functional outcome should be reduced to a non-significant odds ratio [15].

In patients with missing FIVs, values of infarcted volumes were imputed based on relevant baseline covariates, allocated treatment and functional outcome [18]. Due to a skewed distribution of FIV measurements, the confidence interval of the difference of measured median FIVs between both treatment groups was constructed by bootstrapping with 1,000 replications. For the same reason, FIV was transformed to $\sqrt[3]{FIV}$ to achieve linearity for linear regression. Pathway A was tested with linear regression. Pathways B, C and A-B were tested with proportional odds regression without and with adjustments for age, sex, previous diabetes mellitus, previous ischaemic stroke, atrial fibrillation, NIHSS at baseline, occluded internal carotid artery terminus (ICA-T) occlusion, collateral status at baseline CTA, treatment with intravenous alteplase and time from stroke onset to randomisation. Effect estimates were presented as common odds ratios and betas with corresponding 95% confidence intervals (CIs). To assess the proportion of the effect of intervention on functional outcome that was mediated by FIV, the log odds ratio of the indirect effect of intervention in pathway A-B was divided by the log odds ratio of the direct effect of intervention in pathway C [19, 20]. The CIs for the proportion of the effect mediated were constructed with bootstrapping with 1,000 replications. In this approach, the 95% CI can exceed 0% and 100% but we manually truncated the lower bound to 0% and the upper bound to 100%.

All analyses were performed in R statistical software (version 3.4.2) with the packages foreign, rms, gvlma and boot.

### Sensitivity analysis

To test the robustness of our findings against the assumptions that were made, we performed two sensitivity analyses. First, in order to account for patients who died within a week and therefore did not have a NCCT at 5–7 days and for patients who had no NCCT before hemicraniectomy, we imputed FIV in these patients with single imputation. Second, we assessed the effect of replacing missing FIV with FIV assessed from NCCT scans acquired at 24 h.

## Results

### Descriptives

**Fig. 3:**
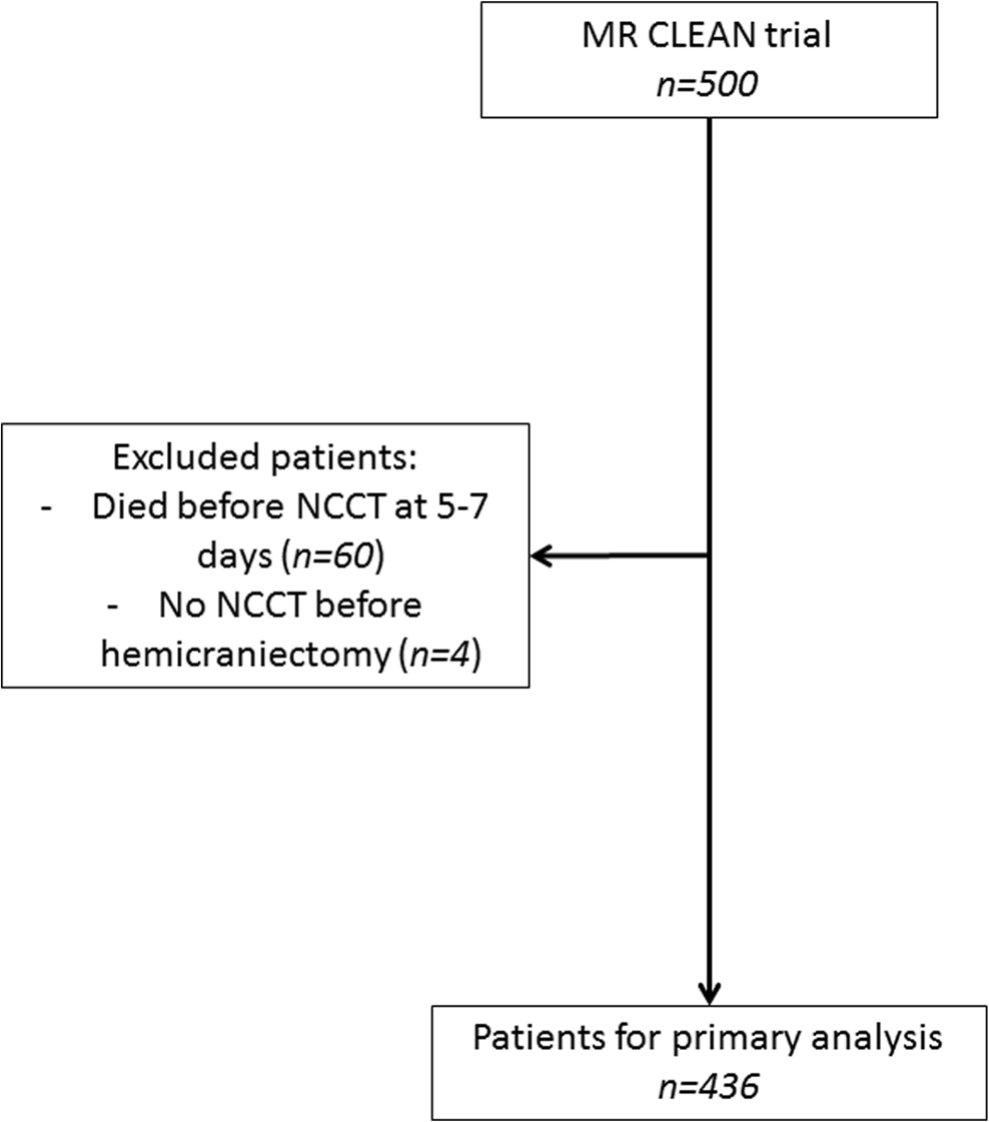
Flowchart of included patients in the primary analysis

In total, 500 patients were included in the MR CLEAN trial. Sixty patients died before the NCCT scan at 5–7 days after initial treatment could be performed and in four patients no NCCT was performed before hemicraniectomy, leaving 436 patients for analysis (Fig. 3). In 99 (23%) of these 436 patients no NCCT scan was made within 1 week because of logistic reasons (n=91) such as transfer back to referring primary stroke centre or hospital discharge or no FIV measurement could be done because of poor scan quality (n=8). Baseline characteristics of analysed patients in both treatment groups are shown in Table 1, median measured FIV in all patients was 67 ml (IQR 30–124). Imaging outcomes regarding reperfusion on digital subtraction angiography (DSA) and recanalisation on follow-up CTA (24 h) of analysed patients are presented in Tables 2 and 3.

**Table 1:** Baseline characteristics of analysed patients (n=436)

	Intervention group (n=198)	Control group (n=238)	Excluded patients (n=64)
Sex (men) (%)	117 (59.1)	134 (56.3)	41 (64.1)
Age (y, median [IQR])	63.47 [53.3 - 73.4]	65.67 [55.1 - 76.4]	71.40 [61.8 – 79.9]
Pre-stroke mRS≤2 (%)	191 (96.5)	229 (96.2)	59 (92.2)
NIHSS at baseline (median [IQR])	17.00 [14.0 - 20.8]	17.00 (14.0 - 21.8)	21.00 [17.0 – 23.0]
Treatment with intravenous alteplase (%)	177 (89.4)	216 (90.8)	52 (81.2)
Time from onset to randomisation (median [IQR])	200.00 [150.0 - 250.0]	194.00 [148.8 - 266.8]	212.00 [174.0 – 258.3]
Smoking (%)	59 (29.8)	72 (30.3)	12 (18.8)
Diabetes (%)	23 (11.6)	29 (12.2)	16 (25.0)
Atrial fibrillation (%)	54 (27.3)	65 (27.1)	16 (25.0)
Previous stroke (%)	23 (11.6)	20 (8.4)	11 (17.2)
Location of intracranial occlusion (%) #			
ICA	1 (0.5)	3 (1.3)	-
ICA terminus	50 (25.3)	65 (27.3)	19 (29.7)
M1	129 (65.2)	146 (61.6)	44 (68.8)
M2	17 (8.6)	21 (8.9)	1 (1.6)
A2	1 (0.5)	2 (0.8)	-
ASPECTS ≥ 8 (%) §	150 (75.0)	194 (82.6)	32 (50.8)

**§** Alberta Stroke Program Early Computed Tomography Score (ASPECTS) ranges from 0 to 10, with higher scores indicating fewer early ischaemic changes. Data were missing for three patients in the control group

**#** Location of intracranial occlusion could not be assessed in one patient in the control group due to non-performed vessel imaging

*IQR* interquartile range

**Table 2:** Explained proportions and effect sizes in the mediation analyses of the effect of intervention on functional outcome mediated by follow-up infarct volume (FIV)

Pathway*		Unadjusted analysis				Adjusted analyses					
		Effect parameter	Primary analysis (n=436)		Effect parameter	Primary analysis (n=436)		Sensitivity analysis 1 (n=500)		Sensitivity analysis 2 (n=436)	
			Value	(95% CI)		Value	(95% CI)	Value	(95% CI)	Value	(95% CI)
A	( X → M )	Beta	-0.34	(-0.64 – -0.05)	Beta	-0.37	(-0.65 – -0.09)	-0.21	(-0.49 – 0.06)	-0.38	(-0.64 – -0.11)
B	( M → Y )	cOR	0.59	(0.52 – 0.66)	acOR	0.58	(0.51 – 0.66)	0.63	(0.56 – 0.70)	0.49	(0.42 – 0.56)
C	( X → Y )	cOR	2.22	(1.58 – 3.13)	acOR	2.30	(1.62 – 3.26)	1.78	(1.29 – 2.46)	2.30	(1.62 – 3.26)
A-B	( X + M → Y)	cOR	2.03	(1.44 – 2.86)	acOR	2.05	(1.44 – 2.91)	1.66	(1.20 – 2.30)	2.04	(1.43 – 2.90)
ß _FIV_^#^		OR	0.60	(0.53 – 0.67)	OR	0.60	(0.52 – 0.67)	0.66	(0.58 – 0.74)	0.49	(0.43 – 0.56)
Explained proportion		%				14	(0 – 34)	12	(0 – 43)	15	(0 - 38)

*Each pathway is shown in Fig. 1. X, independent variable (intervention- or control group). M, mediator variable (follow-up infarct volume), Y, dependent variable (functional outcome)

# ß, coefficient of FIV (mediator) in pathway A-B must be a significant predictor of the dependent variable, while controlling for the independent variable

*cOR* common odds ratio, *acOR* adjusted common odds ratio, 95% CI, 95% confidence interval

**Table 3:** Imaging outcomes regarding reperfusion on DSA after intervention

	Intervention group (n=166)		Control group (NA)
Reperfusion grades on DSA (%) and median follow-up infarct volumes (ml) [IQR] ^#^			
0	18 (11)	107 [58-246]	-
1	9 (5)	137 [62-222]	-
2a	32 (19)	55 [30-118]	-
2b	64 (39)	41 [21-93]	-
3	43 (26)	56 [35-105]	-

^#^Assessed by the modified Thrombolysis in Cerebral Infarction (mTICI): 0 – no reperfusion, 1 – antegrade flow past the initial occlusion, but limited distal branch filling with little or slow distal reperfusion, 2a – antegrade reperfusion of less than half of the previously ischaemic territory, 2b – antegrade reperfusion of more than half of the previously ischaemic territory, 3 – complete antegrade reperfusion of the previously ischaemic territory, with absence of visualised occlusion in all distal branches. Scores were not available for 32 (19%) patients in the intervention group

### Mediation analysis

In step 1 of the mediation analysis, we tested the relationship between intervention and functional outcome. Treatment was indeed a significant predictor of functional outcome. In the present dataset, the adjusted common odds ratio (acOR) was 2.30 (95% CI 1.62–3.26). In step 2, we tested the relationship between allocated treatment and FIV. The median FIV was 53 ml (IQR 24–116) in the intervention group and 81 ml (IQR 35–127) in the control group (difference 28 ml; 95% CI 13–41). Intervention was significantly related to reduction in transformed FIV with a beta of -0.37 (95% CI -0.65 – -0.09). In step 3, we tested the relationship between FIV and functional outcome, with adjustment for treatment allocation. The mediator FIV was an independent variable and a significant predictor of functional outcome with cOR of 0.60 (95% CI 0.52–0.67) per 10 ml. The direct effect of intervention on functional outcome remained statistically significant after adjustment for FIV with an acOR 2.05 (95% CI 1.44–2.91) (Table 4). We found that preventing progression of FIV explains 14% (95% CI 0–34) of the beneficial effect of intervention on functional outcome. All unadjusted estimates were comparable to adjusted estimates.

**Table 4:** Imaging outcomes regarding recanalisation on CTA at 24-hours

	Intervention group (n=173)		Control group (n=190	
Recanalisation status on follow up CTA (%) and median follow-up infarct volumes (mL) [IQR] ^#^				
0	16 (9%)	200 [65-324]	59 (31%)	93 [48-141]
1	4 (2%)	168 [104-231]	18 (9%)	103 [82-126]
2	15 (9%)	24 [12-61]	46 (24%)	82 [33-117]
3	138 (80%)	47 [23-86]	67 (35%)	51 [19-82]

^#^Assessed by the modified Arterial Occlusive Lesion (mAOL) score on CTA at 24 h: 0 – no recanalisation of primary intracranial occlusion, 1 – incomplete or partial recanalisation of the primary intracranial occlusion without contrast passage, 2 – incomplete or partial recanalisation of the primary intracranial occlusion with contrast passage, 3 – complete recanalisation of the primary intracranial occlusion. Values were missing for 25 (13%) patients in the intervention group and 48 (20%) patients in the control group

*IQR* interquartile range

### Sensitivity analysis

In the first sensitivity analysis, including all patients, 35 patients in the intervention group and 29 patients in the control group who died within 1 week or underwent hemicraniectomy before NCCT were additionally included by single imputation. The results of the steps were consistent with the primary analysis (Table 4). The proportion of explained mediated effect was 12% (95% CI 0–43). In the second sensitivity analysis, the missing 5–7 days NCCT FIV were replaced by 24-h NCCT if performed instead of imputation (leaving 30 missing FIVs) and resulted in an explained mediated effect of 15% (95% CI 0–38).

## Discussion

In this study, we tested with mediation analysis whether the beneficial effect of intervention for acute ischaemic stroke on functional outcome could be explained by FIV. We found that FIV on NCCT at 5–7 days was affected by treatment, and was related to functional outcome, but only explained a modest part of the effect of intervention on functional outcome at 90 days measured by the modified Rankin scale in patients with acute ischaemic stroke. This implies that FIV on NCCT only partially explains the effect of intervention on functional outcome and should therefore not be used as an early surrogate imaging marker for clinical endpoints in trials.

A previous study found a significant association between volume of FIV on NCCT and three different functional outcome measurements at 3 months. However, a moderate correlation between infarct volume and all functional outcome measures was found [11]. The study did not report the commonly used mRS score as a functional outcome measurement. Another study, which also included ischaemic stroke patients undergoing intervention, demonstrated that FIV was an important determinant of functional outcome at 3 months [4]. However, this study used imaging (NCCT or MRI) in a broad time window between 24 h and 2 weeks after stroke. Our conclusion also differs from a previous study on this topic, which concluded that FIV explains the effect of intervention on functional outcome [6]. However, in that study only the first and second step of mediation analysis were performed, and not the third step. This implies that no definite conclusion on mediation could be drawn, which explains the discrepancy with our findings. No other studies that reported an association between FIV and functional outcome did not perform a full a causal mediation analysis. In the REVASCAT study a mediation analysis was carried out, with similar results, but the proportion of explained treatment effect was not estimated [16]. Our study is the first full mediation analysis to analyse the pathway from intervention to FIV to functional outcome and report the proportion of explained treatment effect mediated by FIV.

Several assumptions must be made to perform an unbiased causal mediation analyses [21]. First, there is no unmeasured confounding between treatment and outcome; this assumption is automatically satisfied in our study due to randomisation of treatment. Secondly, no unmeasured confounding between mediator and outcome should be present. This is true for our study as the observers were blinded with respect to clinical information during imaging analysis [9]. Third, there should be no unmeasured confounding between the treatment and mediator. This requirement is also satisfied in our study due to randomisation and the fact that FIV measurements were assessed after baseline. This is also confirmed by the consistency of the results of the adjusted and unadjusted analyses.

A limitation of our study is the exclusion of deceased patients in the first week after onset and therefore missing FIV measurements at 5- to 7-day follow-up (n=60 (12%)). In our sensitivity analysis, we tried to overcome this by imputing FIVs in these deceased patients. Results of the sensitivity analysis did not change the conclusions of our paper and effect sizes are comparable. Although factors other than FIV possibly play a role in early death, it is likely that the more severely affected patients with potentially large FIV will be over-represented among patients who died early [4, 6]. In our study, no FIV measurements on NCCT in 99 patients could be assessed at 5- to 7-day follow-up mostly because of logistic reasons; this could result in a distortion of the results. We therefore used imputation techniques to adjust for this potential bias [22, 23]. The estimates of the mediator (FIV measurements) must be reliable and valid. Our automated, observer-checked estimation method has been shown to be reliable [17]. Overestimation of infarct size due to oedema may occur. The randomised assessment of treatment effect will reduce this bias. In a sensitivity analysis, we showed that use of 24-h NCCT FIV for missing FIV did not increase the explained proportion, probably because FIV measurement is less precise, and hypodense areas may yet increase in size.

Another limitation is our relatively small sample size. The different pathways in our mediation model (EVT-FIV, FIV-functional outcome and EVT- functional outcome) are all frequently studied and confirmed in multiple datasets. However, the proportion of the effect of EVT on functional outcome has never been calculated before. Our relatively wide CI expressed the uncertainty in this estimate. Therefore, our findings need to be replicated in other randomised control trials performed on EVT.

Our study made use of follow-up NCCT to assess FIV, because this is the most widely available and used modality. It would be of interest to also study effect mediation by FIV measured with MRI. Care should be taken, however, that selection bias in assessment does not distort the comparison between MRI and CT.

A large proportion of the treatment effect on functional outcome remains unexplained, suggesting FIV alone cannot be used as an early proxy of functional outcome. Effects of other pathways may play a role in determining functional outcome such as infarct location. Previous studies have demonstrated that certain brain regions are more sensitive than others to hypoperfusion, which may interact with FIV regarding functional outcome, given that the relevance for functional outcome varies by regional eloquence [24–26]. In our study, patients had an occlusion of the middle cerebral artery supplying eloquent brain regions. Small lesions in eloquent regions may have a larger destructive effect on functional outcome than larger infarcts in non-eloquent regions.

Further studies should address the question whether combining FIV with a measure of eloquence can increase the predictive value for functional outcome [26, 27]. Taking eloquence into account might improve the proportion of explained mediated effect. The best method to combine eloquence, location and infarct volume is not yet known. Infarcts do not only affect the cortical regions but also white matter tracts. Small infarcts in eloquent cortical regions or important white matter tracts might result in severe strokes. This type of analysis, which takes into account the location of infarct in mediation models, requires larger datasets. In our study, we were mainly interested in FIV as a surrogate imaging biomarker as a first step, because it has been used in several studies [28]. However, for further understanding of the pathophysiological mechanisms relating infarct volume to functional outcome, taking location into account is the obvious next step [26]. Another approach could be to combine the FIV measurement with assessments of specific stroke symptoms and stroke severity. NIHSS is currently increasingly used for assessment of initial stroke severity in clinical practice [29–31], but it might also be an interesting intermediate outcome measurement [16]. Also, in our primary analysis, we assessed FIV at 5–7 days. FIV on NCCT in other time windows might also be of interest as a surrogate marker [32]. Finally, the use of more advanced imaging modalities such as MRI to determine FIV would be interesting for future studies.

In conclusion, we confirmed that intervention prevents progression of FIV on NCCT, but this only partly explains the beneficial effect of intervention on functional outcome.

## Electronic supplementary material

ESM 1 (DOCX 16 kb)

## Abbreviations

CT: Computed tomography
CTA: Computed tomography angiography
DSA: Digital subtraction angiography
EVT: Endovascular treatment
FIV: Follow-up infarct volume
MR CLEAN: Multicenter Randomized Clinical Trial of Endovascular Treatment for Acute Ischemic Stroke in The Netherlands
MRA: Magnetic resonance angiography
MRI: Magnetic resonance imaging
mRS: Modified Rankin Scale
NCCT: Non-contrast computed tomography
NIHSS: National Institutes of Health Stroke Scale
