# Supplementary material for: Follow-up infarct volume as a mediator of endovascular treatment effect on functional outcome in ischaemic stroke
Source: Eur Radiol. 2018 Jul 9;29(2):736–44. doi: 10.1007/s00330-018-5578-9 (PMC6302877; doi:10.1007/s00330-018-5578-9)
Supplement: Supplementary file 1 — (DOCX 16 kb) [file 330_2018_5578_MOESM1_ESM.docx]

Appendix (Suppl. Tab. 1)

**MR CLEAN Investigators**

Executive Committee

Diederik W.J. Dippel (Department of Neurology, Erasmus MC University Medical Center); Aad van der Lugt (Radiology, Erasmus MC University Medical Center); Charles B.L.M. Majoie (Department of Radiology, Academic Medical Center, Amsterdam); Yvo B.W.E.M. Roos (Neurology, Academic Medical Center, Amsterdam); Robert J. van Oostenbrugge (Department of Neurology, Maastricht University Medical Center and Cardiovascular Research Institute Maastricht (CARIM)); Wim H. van Zwam (Radiology, Maastricht University Medical Center and Cardiovascular Research Institute Maastricht (CARIM)); Olvert A. Berkhemer (Department of Neurology, Erasmus MC University Medical Center; Department of Radiology, Academic Medical Center, Amsterdam); Puck S.S. Fransen (Department of

Neurology and Radiology, Erasmus MC University Medical Center); Debbie Beumer (Department of Neurology, Erasmus MC University Medical Center; Department of Neurology, Maastricht University

Medical Center and Cardiovascular Research Institute Maastricht (CARIM), the Netherlands); Lucie A. van den Berg (Neurology, Academic Medical Center, Amsterdam)

Local Principal Investigators

Wouter J. Schonewille (Department of Neurology, Sint Antonius Hospital, Nieuwegein, the Netherlands); Jan Albert Vos (Radiology, Sint Antonius Hospital, Nieuwegein, the Netherlands); Charles B.L.M. Majoie (Department of Radiology, Academic Medical Center, Amsterdam, the Netherlands); Yvo B.W.E.M. Roos (Neurology, Academic Medical Center, Amsterdam, the Netherlands); Paul J. Nederkoorn (Neurology, Academic Medical Center, Amsterdam, the Netherlands); Marieke J.H. Wermer (Department of Neurology, Leiden University Medical Center, the Netherlands); Marianne A.A. van Walderveen (Radiology, Leiden University Medical Center, the Netherlands); Robert J. van Oostenbrugge (Department of Neurology, Maastricht University Medical Center and Cardiovascular Research Institute Maastricht (CARIM), the Netherlands); Wim H. van Zwam (Radiology, Maastricht University Medical Center and Cardiovascular Research Institute Maastricht (CARIM), the Netherlands); Julie Staals (Department of Neurology, Maastricht University Medical Center and Cardiovascular Research Institute Maastricht (CARIM), the Netherlands); eannette Hofmeijer (Department of Neurology, Rijnstate Hospital, Arnhem, the Netherlands); Jacques A. van Oostayen (Radiology, Rijnstate Hospital, Arnhem, the Netherlands); Geert J. Lycklama à Nijeholt (Department of Radiology, MC Haaglanden, the Hague, the Netherlands); Jelis Boiten (Neurology, MC Haaglanden, the Hague, the Netherlands); Diederik W.J. Dippel (Department of Neurology, Erasmus MC University Medical Center, the Netherlands); Patrick A. Brouwer (Radiology, Erasmus MC University Medical Center, the Netherlands); Bart J. Emmer (Radiology, Erasmus MC University Medical Center, the Netherlands); Sebastiaan F. de Bruijn (Department of Neurology, HAGA Hospital, the Hague, the Netherlands); Lukas C. van Dijk (Radiology, HAGA Hospital, the Hague, the Netherlands); L. Jaap Kappelle (Department of Neurology, University Medical Center Utrecht, the Netherlands); Rob H. Lo (Radiology, University Medical Center Utrecht, the Netherlands); Ewoud J. van Dijk (Department of Neurology, Radboud University Medical Center, Nijmegen, the Netherlands); Joost de Vries (Neurosurgery, Radboud University Medical Center, Nijmegen, the Netherlands); Paul L.M. de Kort (Department of Neurology, Sint Elisabeth Hospital, Tilburg, the Netherlands); Jan S.P. van den Berg (Department of Neurology, Isala Klinieken, Zwolle, the Netherlands); Willem Jan J. van Rooij (Department of Neurology, Isala Klinieken, Zwolle, the Netherlands); Boudewijn A.A.M. van Hasselt (Radiology, Isala Klinieken, Zwolle, the Netherlands); Leo A.M. Aerden (Department of Neurology, Reinier de Graaf Gasthuis, Delft, the Netherlands); René J. Dallinga (Radiology, Reinier de Graaf Gasthuis, Delft, the Netherlands); Marieke C. Visser (Department of Neurology, VU Medical Center, Amsterdam, the Netherlands); Joseph C.J. Bot (Radiology, VU Medical Center, Amsterdam, the Netherlands); Patrick C. Vroomen (Department of Neurology, University Medical Center Groningen, the Netherlands); Omid Eshghi (Radiology, University Medical Center Groningen, the Netherlands); Tobien H.C.M.L. Schreuder (Department of Neurology, Atrium Medical Center, Heerlen, the Netherlands); Roel J.J. Heijboer (Radiology, Atrium Medical Center, Heerlen, the Netherlands); Koos Keizer (Department of Neurology, Catharina Hospital, Eindhoven, the Netherlands); Alexander V. Tielbeek (Radiology, Catharina Hospital, Eindhoven, the Netherlands); Heleen M. den Hertog (Department of Neurology, Medical Spectrum Twente, Enschede, the Netherlands); Dick G. Gerrits (Radiology, Medical Spectrum Twente, Enschede, the Netherlands); Renske M. van den Berg-Vos (Department of Neurology, Sint Lucas Andreas Hospital, Amsterdam, the Netherlands); Giorgos B. Karas (Radiology, Sint Lucas Andreas Hospital, Amsterdam, the Netherlands)

Imaging Assessment Committee

Charles B.L.M. Majoie (Chair, Department of Radiology, Academic Medical Center, Amsterdam, the Netherlands); Wim H. van Zwam (Radiology, Maastricht University Medical Center and Cardiovascular Research Institute Maastricht (CARIM), the Netherlands); Aad van der Lugt (Radiology, Erasmus MC University Medical Center, the Netherlands); Geert J. Lycklama à Nijeholt (Department of Radiology, MC Haaglanden, the Hague, the Netherlands); Marianne A.A. van Walderveen (Radiology, Leiden University Medical Center, the Netherlands); Joseph C.J. Bot (Radiology, VU Medical Center, Amsterdam, the Netherlands); Henk A. Marquering (Biomedical Engineering and Physics, Academic Medical Center, Amsterdam, the Netherlands); Ludo F. Beenen (Department of Radiology, Academic Medical Center, Amsterdam, the Netherlands); Marieke E.S. Sprengers (Department of Radiology, Academic Medical Center, Amsterdam, the Netherlands); Sjoerd F.M. Jenniskens (Radiology, Radboud University Medical Center, Nijmegen, the Netherlands); René van den Berg (Department of Radiology, Academic Medical Center, Amsterdam, the Netherlands); Olvert A. Berkhemer (Department of Neurology, Erasmus MC University Medical Center; Department of Radiology, Academic Medical Center, Amsterdam, the Netherlands); Albert J. Yoo (Department of Radiology, Texas Stroke Institute)

Outcome Assessment Committee

Yvo B.W.E.M. Roos (Chair, Neurology, Academic Medical Center, Amsterdam, the Netherlands); Peter J. Koudstaal (Department of Neurology, Erasmus MC University Medical Center, the Netherlands); Jelis Boiten (Department of Neurology, Radboud University Medical Center, Nijmegen, the Netherlands); Ewoud J. van Dijk (Department of Neurology, Radboud University Medical Center, Nijmegen, the Netherlands)

Adverse Event Committee

Robert J. van Oostenbrugge (Chair, Department of Neurology, Maastricht University Medical Center and Cardiovascular Research Institute Maastricht (CARIM), the Netherlands); Marieke J.H. Wermer (Department of Neurology, Leiden University Medical Center, the Netherlands); H. Zwenneke Flach (Radiology, Isala Klinieken, Zwolle, the Netherlands)

Trial Statisticians

Ewout W. Steyerberg (Public Health, Erasmus MC University Medical Center, the Netherlands; Medical Statistics and Bioinformatics, Leiden University Medical Center, the Netherlands); Hester F. Lingsma (Public Health, Erasmus MC University Medical Center, the Netherlands); Medical Statistics and Bioinformatics
